# Breaking the 100-nm resolution barrier with multiphoton microscopy using image scanning microscopy and optical fluctuation imaging

**DOI:** 10.1117/1.JBO.31.8.086501

**Published:** 2026-08-02

**Authors:** Anton Classen, Alma Fernández, Ajithamithra Dharmasiri, Cristobal Rodriguez, Rahul Srinivasan, Aleksei M. Zheltikov, Girish S. Agarwal, Aart J. Verhoef

**Affiliations:** a Texas A&M University, Department of Soil and Crop Sciences, College Station, Texas, United States; b University of Utah, Core Research Facilities, Salt Lake City, Utah, United States; c Texas A&M University, Department of Physics and Astronomy, College Station, Texas, United States; d Texas A&M University, Institute for Quantum Science and Engineering, College Station, Texas, United States; e Texas A&M University, Department of Neuroscience & Experimental Therapeutics, Bryan, Texas, United States; f Texas A&M University, Department of Biological and Agricultural Engineering, College Station, Texas, United States

**Keywords:** multiphoton microscopy, super-resolution, image scanning microscopy, optical fluctuations, SOFI.

## Abstract

**Significance:**

Multiphoton fluorescence microscopy is the technique of choice for investigations of thick, highly scattering samples, but is outperformed by single-photon super-resolution techniques in spatial resolving power.

**Aim:**

We combine two-photon microscopy with two super-resolution microscopy methods, namely, image scanning microscopy and super-resolution optical fluctuation imaging to overcome the reduction in resolution of laser scanning multiphoton microscopy compared with confocal microscopy. Making use of higher-order cumulants and image deconvolution a resolution better than 100 nm can be achieved.

**Approach:**

Two-photon image scanning optical fluctuation imaging is achieved by detecting the descanned signal of fluorescence on a 23-element single photon avalanche detector and analyzing (higher order) cumulants of the temporal evolution of the signals. We test the performance of our method with samples of dispersed quantum dots. We show the applicability of two-photon image scanning optical fluctuation imaging to biological samples with fixed mouse ventral midbrain neurons with quantum dot labeled tubulin.

**Results:**

Combining two photon laser scanning microscopy with image scanning microscopy allows to overcome the reduction in resolution caused by the longer wavelength excitation inherent to multiphoton excitation. Analyzing the temporal fluctuations of the signals by calculating cumulants allows to surpass the resolution achieved with conventional confocal imaging of the same fluorophores, and the use of higher-order cumulants and deconvolution allows to achieve a lateral resolution of 75 nm when imaging quantum dots emitting at 625 nm.

**Conclusions:**

Combining two-photon microscopy with image scanning microscopy and optical fluctuation imaging allows to achieve a 5-fold improvement in resolution over standard two-photon microscopy, and a 3.5-fold improvement over conventional widefield imaging of the same fluorophores. This represents the first time, to our knowledge, that sub-100 nm imaging is achieved using multiphoton laser scanning microscopy.

## 1 Introduction

Fluorescence microscopy has become one of the most widely used tools to visualize biological processes, imaging of living cells, tissues, and organisms. However, compared with techniques such as electron microscopy (EM), conventional fluorescence microscopy is constrained by relatively low spatial resolution due to the diffraction of light. In conventional far-field fluorescence microscopy the diffraction limit prevents details that are smaller than about half the emission-wavelength (∼200 to 300 nm) to be properly resolved. These limitations had prompted the development of a variety of techniques that have enabled imaging resolutions smaller than the diffraction limit.^1^^–^^12^ Those include concepts such as stimulated emission depletion (STED)^1^ and single molecule localization microscopy (SMLM) such as photo-activated localization microscopy (PALM)^2^ and stochastic optical reconstruction microscopy (STORM).^3^ These techniques have demonstrated remarkable resolution improvement down to less than a nanometer,^4^^,^^5^ but at the expense of more complicated experimental realizations and more involved labeling conditions, such as in the case of STED, and slow acquisition speeds (minutes to hours) for the case of PALM and STORM. Other super-resolution techniques allow for a more modest resolution enhancement (around 2-fold below the diffraction limit).^6^^–^^9^ Those include computational and statistical fluctuation methods^13^ such as second-order super-resolution optical fluctuation imaging (SOFI),^7^ super-resolution radial fluctuations (SRRF),^14^ and photobleaching microscopy^15^ (PiMP), as well as classical optics approaches such as structured illumination microscopy (SIM)^8^^,^^16^ and image scanning microscopy (ISM),^11^^,^^17^ which can be considered a point-scanning variant of SIM.

Techniques such as SIM and ISM have the advantage of being highly compatible with all fluorescent labels, work well with a great variety of samples, and can be implemented in existing microscope systems, without the added complexity of SMLM or STED. SOFI in conventional wide field microscopy has been demonstrated without additional hardware modifications to the microscope setup.^7^ Although SOFI relies on some amount of fluorescence fluctuations, the required photo switching dynamics are far less stringent compared with SMLM. It provides 3D super-resolution enhancement by calculating higher-order cumulants from the spatiotemporal fluctuations of blinking fluorophores in the sample.^7^^,^^18^ Theoretically, the nth order cumulant directly shrinks the PSF √n-fold, and additional Fourier-reweighing or deconvolution (DCV) can boost the final resolution to a full n-fold enhancement. Yet, in practice, higher-order cumulants require longer measurement times, exhibit reduced signal to noise ratios (SNR) as compared with the second order cumulant, and can suffer from cusp artifacts.^19^

Additional resolution enhancement can be achieved by combining complementary methods (e.g., up to 2×2 when combining ISM and SOFI).^18^ Combining SOFI with SIM was theoretically introduced in the work of Classen et al.^20^ and Zhao et al.^21^ SOFI was combined with ISM, in a proof-of-principle experiment, in a technique called SOFISM,^18^ in which the spontaneous blinking nature of sparse quantum dot (QD) samples was utilized to achieve a total resolution enhancement of 2.6 for second-order SOFISM, with a 3  μm by 3  μm field of view. SIM and iSIM^22^ combined with self-blinking dyes for SOFI was presented in Ref. 23 where up to 2.4-fold image resolution increase was obtained. For second-order iSIM-SOFI analysis a field of view of 135  μm by 135  μm was imaged and acquisition times ranged from 20 to 50 s (200 to 500 frames). Analyzing 3500 frames allowed to perform up to third-order SOFI analysis, but due to fixed pattern artifacts in the cumulant processed images, the authors were prevented from determining proper resolution estimates.^23^

For imaging thicker highly scattering biological samples, multiphoton imaging has become the gold standard,^24^^–^^26^ but it provides lower spatial resolution than single photon (1PE) confocal laser scanning microscopy (CLSM). Two-photon-excitation ISM (2PE-ISM),^27^^–^^29^ and more recently three-photon ISM (3PE-ISM)^6^ have been demonstrated to enable in-depth super-resolution imaging in thicker samples and scattering tissues, that cannot be studied with 1PE alone. Similarly, multiphoton imaging has been combined with SOFI to achieve higher resolution deep imaging. This was achieved in an implementation using wide-field excitation, applied via a technique called temporal focusing (TF).^30^ TF allows for fast image acquisition and achieves reduced photo-toxicity and photo-bleaching compared with 1PE widefield imaging; however, the high pulse energy requirement can induce unwanted thermal effects as discussed in Ref. 31.

Hybridizing multiphoton-ISM approaches with the power of SOFI will allow for achieving further improvements in spatial resolution. The ISM and SOFI techniques were previously combined, in a microscope setup based on 1PE CLSM.^18^ Here, we experimentally demonstrate a hybrid technique that combines 2PE-ISM with SOFI. There are two main experimental strategies for image acquisition and analysis. First, a slow scanning approach with long dwell times (30 to 100 ms), in which the SOFI cumulant correlation analysis is applied directly on neighboring SPAD pixel time traces, before the ISM shift processing, similar to the approach dubbed SOFISM.^18^ In our experiments, we found that long pixel dwell times with two-photon excitation often drives the quantum dots into a dark state that does not recover within the dwell time, making this approach less practical. The second approach employs fast scanning of the laser spot multiple times over the field-of-view to obtain a frame series. Here, we first assemble the ISM image for each time frame, followed by SOFI analysis on the assembled ISM frames, which is similar to SOFI analysis on wide-field fluorescence images. We refer to this second approach as ISM-SOFI. We found that this approach, the ISM-SOFI method, allows for reducing sample damage and avoids measurement artifacts observed in 2P-SOFISM. This approach has also been applied in the iSIM-SOFI demonstration.^23^ We were able to image samples of blinking QD dispersed on a glass slide as well as fixed cell sample labeled with blinking QDs. 2PE-ISM provides a resolution enhancement of 1.3 over conventional 2PE microscopy (which provides roughly 370 nm resolution in our setup), and (2nd order) SOFI processing enhances the resolution by an additional factor of 1.4, to achieve a combined enhancement of 1.8. Adding Richardson–Lucy deconvolution boosts the total resolution enhancement factor to 3 (∼120  nm). Higher-order SOFI processing allows for further resolution enhancement. Fourth order SOFI processing allows for a more than two-fold improvement over the 2PE-ISM resolution, and deconvolution pushes the resolution to well below 100 nm.

## 2 ISM-SOFI Theory and Data Processing

The ISM imaging approach starts with point-scanning focused laser excitation, and a small pixel array located at the position of the pinhole in confocal microscopy. For the SPAD23 detector (Pi Imaging Technologies), 23 detection pixels at relative positions $s_{j}$ around the central pixel capture the emitted fluorescence. The central pixel acts as a very small on-axis confocal pinhole, whereas the surrounding pixels act as very small off-axis pinholes whose signal needs to be reassigned to obtain the ISM resolution enhancement. In our configuration, and at emission wavelength of around $\lambda_{\det}=625\;\mathrm{nm}$ each pixel is around 0.2 AU in size. For these almost closed pinholes, we can use the approximation.^17^

$$\mathrm{PSF}(r,s_{j})={\mathrm{PSF}}_{\mathrm{exc}}(r)*{\mathrm{PSF}}_{\det}(r-s_{j}),$$

where the two-photon excitation PSF reads ${\mathrm{PSF}}_{\mathrm{exc}}(r)=[(2J_{1}(r)/r{)}^{2}{]}^{2}$, with $\lambda_{\mathrm{exc}}=1040\;\mathrm{nm}$. The image captured by each SPAD pixel (j=1,…,23) is the convolution of the fluorescent structure n(r) with the PSF,

$$I_{j}(r)\equiv n(r)⊗\mathrm{PSF}(r,s_{j}).$$

The final ISM image is the sum over all 23 sub-images, shifted by the respective vectors $\alpha s_{j}$.

$$I_{\mathrm{ISM}}(r)=\overset{N}{\underset{i=1}{\sum }}I_{j}(r-\alpha s_{j}).$$

For 2PE-ISM the shift vectors are scaled^6^ by α=0.65. Note that for 1PE with equal excitation and emission wavelengths the shift vector would be scaled by 0.50, i.e., exactly half. For pixel pair distances significantly larger than 1.0 AU, one can devise other pixel shift strategies,^32^ but this is not necessary here.

Then we apply the widefield-like second-order XC-SOFI processing. For each pixel pair combination ($r_{1},r_{2}$) we obtain

$$\text{XC-SOFI}(r_{1},r_{2},\tau =0)=\overset{N}{\underset{i=1}{\sum }}(\Delta \mathrm{ISM}(r_{1},i))*(\Delta \mathrm{ISM}(r_{2},i+\tau )).$$

Note that for second order, the SOFI cumulant over the time trace is identical to the fluctuation-correlations, i.e., the central moments^7^ with ΔISM(r,i)≡ISM(r,i)−⟨ISM(r,i)⟩. The sum is taken over the frame series i=1,…,N. Here, we mainly focus on equal-time correlations with τ=0. Additional τ>0 can be considered, but their contributions drop off^33^ with increasing τ.

Computations were done in a custom MATLAB script. The full 2D+time ISM image series were treated as 3D matrices (X, Y, T) in MATLAB. After the $r_{2}$ shift (noncircular permutation, with values set to zero at the edge) matrix-matrix multiplication followed by summation enabled rapid computation of SOFI cumulants over the frame series. To obtain the final XC-SOFI(r) images, we sum over all evaluated pixel pairs signals (total of 20 here) while shifting each contribution to its true position $(r_{1}+r_{2})/2$. If assuming that $r_{1}\equiv (0,0)$ is always the central pixel, the shift reads $0.5r_{2}$ (where the reassignment vector is scaled by exactly half, because both pixels are of equal weight). Note that we did not include the AC-SOFI signal with $r_{1}=r_{2}$ here.

We utilized pixel-pair cross-correlations (XC-SOFI) without the virtual interpolated pixels of the original XC-SOFI approach.^34^ Instead, we linearly interpolate the calculated cross-correlation signals into the existing pixel grid (but shifted to their true position). This is reminiscent of the ISM shift approach that does not require creation of a virtual, interpolated grid with smaller pixels. This approach also avoids the “flattening” process that is based on “distance factor correction using relative variance minimization”^34^ and which can create residual artifacts.^23^^,^^35^

We included pixel pairs within a meaningful range around the center. This included four direct neighbors (0,1), (+1,0), (0,−1), (−1,0), four direct diagonal neighbors, four direct neighbors at double distance, e.g., (+2,0), and eight 30-deg angle neighbors (e.g., −2, +1), for a total of twenty pixel-pairs for XC-SOFI of second-order. Additional pixel pairs can be included for increased statistics, but they would yield decreased contributions due to a drop off in correlation, due to the reach of the PSF. For any (2,1) pixel pair the distance reads 95 nm, whereas the HWHM of the ISM-PSF is ∼135  nm.

Note that our pixel dimension of 42.5 nm and PSF size (∼370  nm for simple 2P confocal images, versus ∼270 after ISM processing) enabled good oversampling of the signal. After XC-SOFI processing the results are still well within good Nyquist sampling. Hence, the creation of a finer pixel grid and the required flattening were not utilized here. To confirm that XC-SOFI without creation of a new finer pixel grid works well, we tested our XC-SOFI processing script on simulated data, created with the SOFI simulation tool.^36^ It yielded excellent results.

In addition, we considered higher-order XC-SOFI processing. It was possible to remain on the native pixel grid (same as for second-order). The script needed to be modified to include three-point and four-point pixel products, respectively, and then compute the third- and fourth-order cumulants to isolate the higher-order PSF powers. Shift vectors then read $0.33(r_{2}+r_{3})$ and $0.25(r_{2}+r_{3}+r_{4})$. For higher orders, the blinking dynamics (on/off time ratio) needs to be considered. We tested our script with simulated frames using the SOFI simulation tool.^36^ Results of this test are shown in Figs. S1 and S2 in the Supplementary Material. Although second-order SOFI always produced positive results, higher-orders required compensation via the Bernoulli coefficients derived from the on-off time ratio.^18^ For third order, the cumulant is still identical to the third-order central moment. This simplifies the expression and simply requires a sign-flip if the Bernoulli coefficient is negative. For fourth order, the cumulant is a combination of the fourth-order central moment and products of the second-order moments.

We applied an initial 2-fold Fourier interpolation to the XC-SOFI images to accommodate higher resolutions after DCV processing. This reduced the pixel size from 42.5 to 21.25 nm. The initial resolutions of 2PE-ISM-SOFI images can accommodate this interpolation step because they are slightly oversampled with respect to the Nyquist sampling criterion.

To compensate for brightness skewing of second and higher order SOFI signals, brightness linearization steps can be applied to XC-SOFI processed images, similar to the procedure described in Ref. 37. In our procedure, first, 10 iterations of RL (Fiji, DeconvolutionLab2) are applied with the n-th power of the 2PE-ISM PSF for the n-th order XC-SOFI image. Next, we applied the square root to achieve brightness compensation as shown in Fig. S2 in the Supplementary Material. We use a limited number of RL iterations during the initial sharpening step to avoid over-fitting and deconvolution artifacts. A second step deconvolution approaches the maximal expected resolutions for second and higher order cumulant analyses. A two-step deconvolution approach for SOFI has been previously described in Ref. 38, in which the authors utilized initial RL deconvolution to improve image quality of the raw data frame series and then a second DCV step + square root brightness linearization. Although the order is slightly different, resulting image quality and sharpness are improved in both approaches.

## 3 Experimental Realization of 2PE-ISM-SOFI

### 3.1 Microscope

**Fig. 1:**
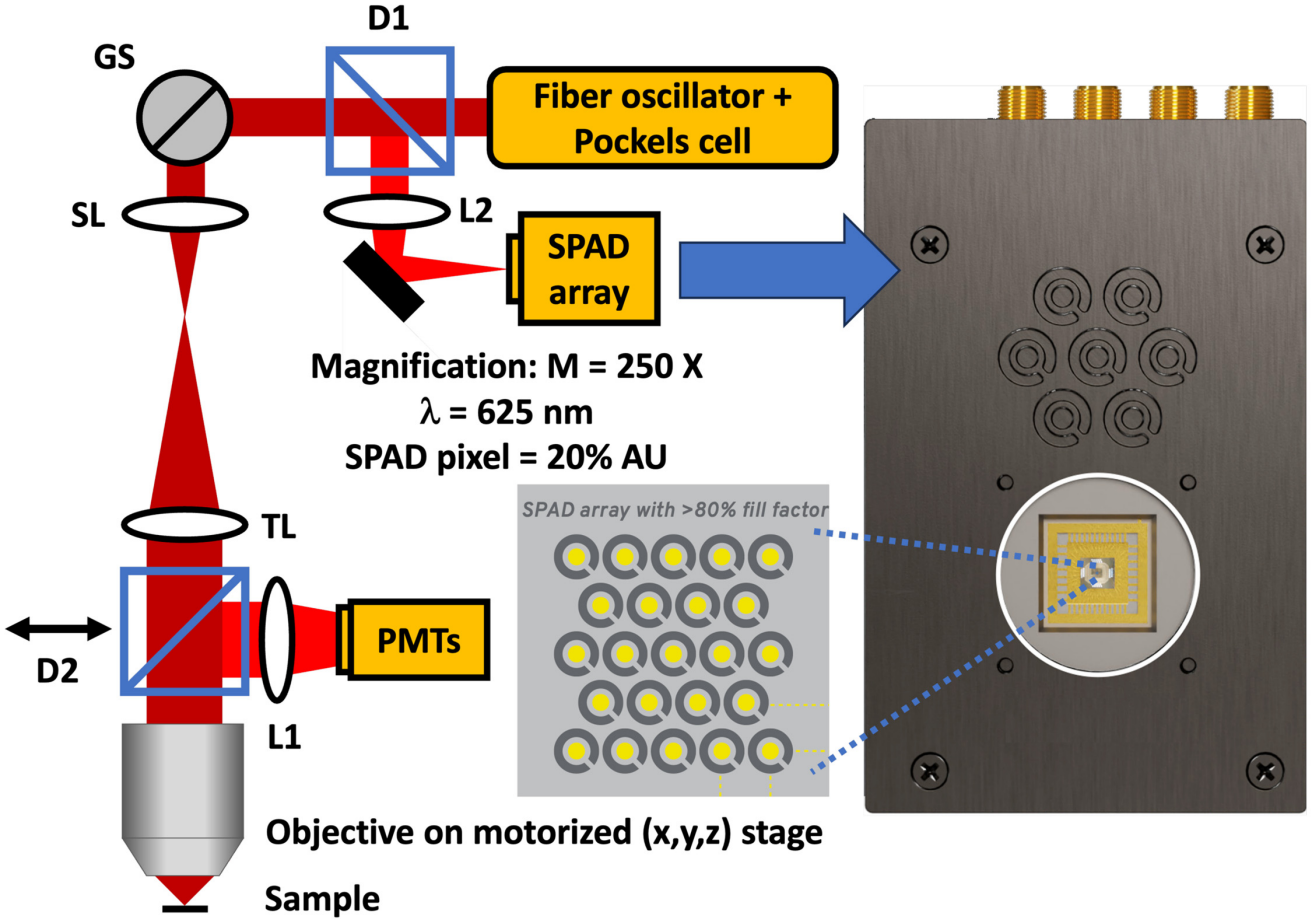
Schematic of the experimental setup. For details see text in Sec. 3.1.

A schematic illustration of the optical setup used for this work is shown in Fig. 1. A high pulse energy Yb-fiber laser is coupled into a customizable modular *in vivo* multiphoton microscope system^39^ as described in Ref. 6. The excitation laser source is a sub-200-fs-pulses in-house-developed Yb:fiber chirped-pulse amplifier with a central wavelength of 1040 nm.^40^ For this work, the system was operated at 4 MHz repetition rate. The beam is focused by a high numerical aperture objective (Nikon MRD71970, 100×, 1.45 NA oil) into the sample. The fluorescence signal is collected by the same microscope objective and imaged with a total magnification of 250× onto a 23-element SPAD array detector (Pi Imaging Technologies) in a descanned detection configuration. Spectral filtering of the desired fluorescent signal is achieved with a dichroic mirror (D1, ZT670rdc-xxrxt, Chroma) placed upstream of the galvanometric mirrors (GS) used for laser-scanning the excitation beam over the sample and descanning the fluorescence signal. An additional red bandpass filter (ET605/70m-2p, Chroma) was placed in front of the SPAD array. The objective is mounted on a three-axis micromanipulator stage (MPC-200, Sutter Instrument) for positioning the objective over the sample and a piezoelectric stage (nPFocus 400, nPoint) for translating the focus axially with high precision (∼10  nm). The fluorescence detection path contains an additional removable long-pass dichroic beamsplitter and band pass filters for “green” (ET525/70m-2p, Chroma) and “red” (ET605/70m-2p, Chroma) for nondescanned imaging with two photomultiplier tube (PMT) detectors. A small sample drift can be noticed when imaging the same area for hundreds of frames, as needed for ISM-SOFI imaging. This small drift is assessed and compensated in postprocessing by performing image registration on successive frames. Because longer pixel dwell times cause unwanted population of longer-lived dark states, we prioritize shorter pixel dwell times and the acquisition of a frame series. We improve the fidelity of image registration and SOFI analysis by averaging several consecutive frames, as the averaged frames have a better SNR. Most robust SOFI analysis is obtained by averaging between 5 and 20 consecutive frames, and for all ISM-SOFI results reported here, we use 10-frame averaging of the raw data before SOFI analysis. However, note that although SMLM approaches require individual isolated single emitter events in the PSF volumes of single frames (and thus 10,000+ frames), the statistical optics approach of SOFI is robust at much higher densities of actively fluorescing emitters within a PSF volume and does not require *a priori* separation of single emitters. This allows SOFI to function with far fewer frames than SMLM. Some approaches even achieve second-order SOFI with as few as 20 frames.^38^^,^^41^ Especially for second order, it was shown that the SOFI SNR stays constant for low, medium, high labeling densities of emitters,^42^ whereas the SNR for higher orders decreases with higher emitter densities within a PSF volume. The XC-SOFI approach here: (1) without virtual pixel creation, with (2) subsequent brightness linearization, and (3) final RL deconvolution produces robust results for as low as 40 input frames, for second and third order SOFI, and 100 frames for fourth order SOFI.

### 3.2 Enhancement over Non-Descanned 2PE Imaging

**Fig. 2:**
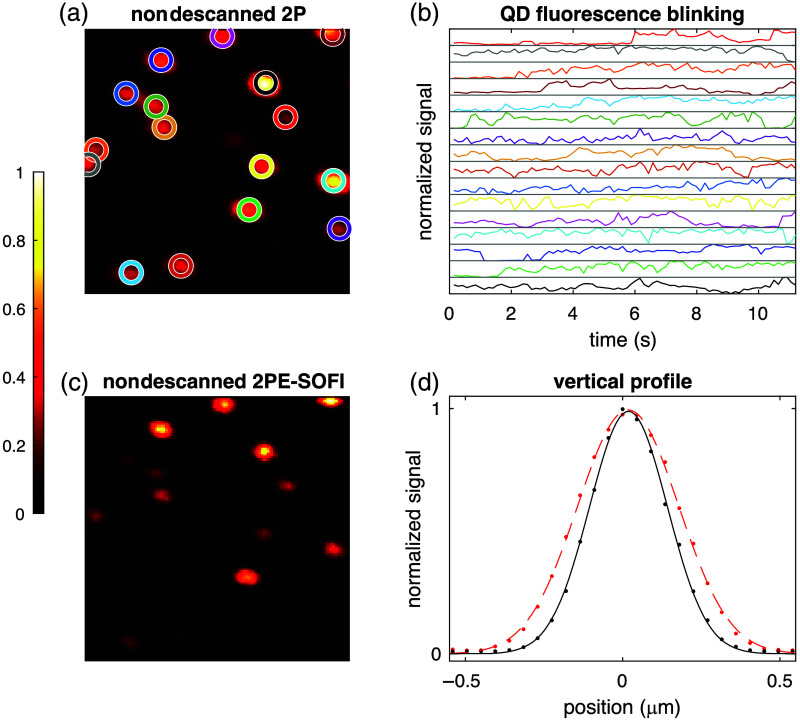
Scanned 2PE-SOFI measurement. (a) Time-averaged image of the nondescanned 2PE fluorescence from the QD625 sample (80 frames, 128×128  pixels, 6.4  μs pixel dwell time). Individual spots are marked by colored circles in the image. (b) Time traces of the signal from individual spots seen in panel (a), integrated over the area indicated by the circles. (c) Result of SOFI processing the temporal evolution of the 2PE images. (d) Average cross-section of 10 nonoverlapping spots in panel (a) dashed red (FWHM 377±5  nm) and panel (c) solid black (FWHM 291±5  nm).

To characterize the resolution improvement of 2PE-ISM-SOFI, we prepare samples of quantum dots fluorescing at 625 nm (QD625) sparsely spread on a microscope coverslip and mounted on a glass slide, similar to the procedure followed in Ref. 18. The QD625 sample (Qdot 625, Thermofisher) was first imaged with two-photon nondescanned detection using the PMT detectors. The resolution of nondescanned 2PE imaging is determined by fitting the width and height of spots corresponding to the time-averaged image of single QDs. SOFI analysis of the time-evolution of the images allows to extract the enhancement of SOFI alone over nondescanned 2PE imaging by fitting the full width at half maximum (FWHM) for the vertical and horizontal cross-sections of the same emitters. Figure 2 shows the results of this measurement. We observe an enhancement for nondescanned 2PE SOFI measurement over nondescanned 2PE imaging of 1.3×, which is only slightly less than the expected theoretical value of 1.41×.

**Fig. 3:**
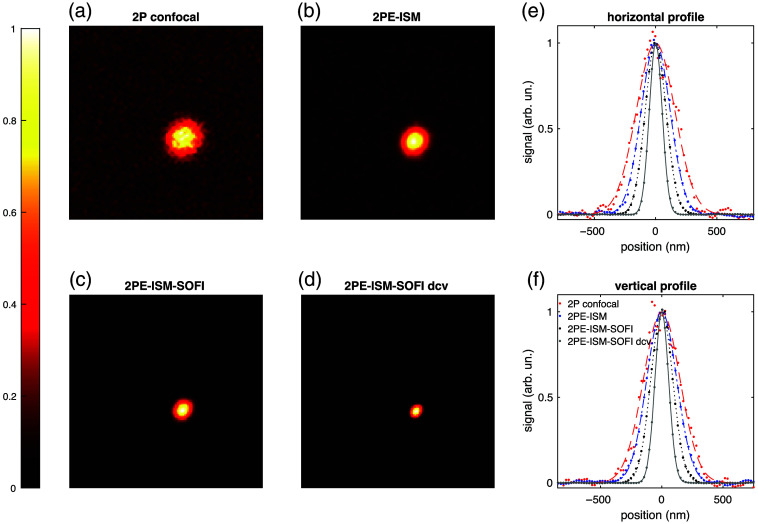
2PE-ISM-SOFI imaging of a single quantum dot. (a) Time-averaged confocal 2P image. (b) Time-averaged 2PE-ISM image. (c) 2PE-ISM-SOFI image. (d) 2PE-ISM-SOFI image with Richardson–Lucy deconvolution. (e), (f) Cross-section of the spot. Color coding: confocal 2P—dashed red (FWHM 371±10  nm), 2PE-ISM—dash-dotted blue (FWHM 277±4  nm), 2PE-ISM-SOFI—dotted black (FWHM 200±1  nm), 2PE-ISM-SOFI dcv—gray (FWHM 123.4±0.4  nm).

Next, the signal was descanned and imaged onto the SPAD array to obtain a time-series of ISM images. From this measurement, we extract the “2PE confocal” resolution (simple sum over all SPAD pixel signals), the 2PE-ISM resolution, and the 2PE-ISM second order SOFI resolution, as shown in Fig. 3. The details of data processing are described in the theory and data processing section. The raw dataset in Fig. 3 consists of 1000 frames of 64×64  pixel images with 64  μs pixel dwell time.

From these results, we calculated the resolution improvement for 2PE-ISM over 2P confocal imaging of 1.34× and a resolution enhancement for 2PE-ISM-SOFI over 2PE-ISM of 1.39×. Thus, the enhancement of 2PE-ISM-SOFI over 2P confocal imaging is 1.85×. Richardson–Lucy deconvolution applied to 2PE-ISM-SOFI, allows for further improvement of 1.62 in the imaging resolution, reaching ∼120  nm resolution.

### 3.3 Imaging with Higher-Order Correlations

The resolution enhancement of SOFI is dependent on the correlation order being evaluated as mentioned above. Higher order SOFI results have a more complex dependence on the on/off-time ratio of the blinking fluorophores than second order SOFI, generally show lower SNR, and can exhibit cusp artifacts.^19^ This is illustrated by reports of instances of experiments with unsuccessful third order SOFI processing but successful fourth order SOFI processing.^18^ To confirm that our MATLAB processing scripts for higher-order XC-SOFI cumulants are working well, we utilized the SOFI simulation tool^36^ to create simulated datasets with different on/off time ratios (see Fig. S1 in the Supplementary Material). The scripts created excellent results for the simulated datasets, and on/off time ratios affected results as expected. For example, a ratio of 0.5 did not yield a nice third-order SOFI cumulant signal, except noise/small fluctuations.

**Fig. 4:**
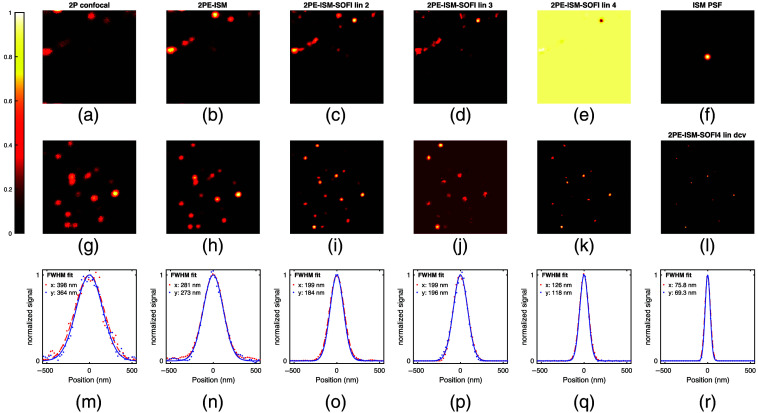
Measurements of QD625 dispersed on a cover slip taken at different excitation power. Top row: 12 mW power at microscope input: (a) 2P confocal image, (b) 2PE-ISM image, (c) linearized ISM-SOFI2 (second order), (d) linearized ISM-SOFI3 (third order), (e) ISM-SOFI4 (fourth order), and (f) calculated ISM PSF from best measurement. Middle row: 6 mW power at microscope input: (g) 2P confocal image, (h) 2PE-ISM image, (i) linearized ISM-SOFI2 (second order), (j) ISM-SOFI3 (third order), (k) linearized ISM-SOFI4 (fourth order), and (l) deconvolved linearized ISM-SOFI4 image. Bottom row: extracted horizontal (dashed red) and vertical (solid blue) profiles. (m) 2P confocal, (n) 2PE-ISM and (o,q) 2PE-ISM-SOFI (second and fourth order), and (r) deconvolved 2P ISM-SOFI4 profiles are extracted from the 6 mW measurement; (p) the 2PE-ISM-SOFI3 (third order) profile is extracted from the 12 mW measurement, which yielded ∼15% larger spotsizes than the 6 mW measurement for the ISM image. The profiles in panels (o)–(r) are taken the ISM-SOFI images before linearization.

For experimental data, we observed that the on/off-time ratio in the case of 2PE of QD625 depends on various factors, including excitation power, pixel dwell time, and frame scan rate. This allowed us to experimentally assess the resolution enhancement for second order, third order, and fourth order SOFI. Figure 4 shows images and cross-sections of quantum dot samples imaged with different acquisition parameters allowing comparison of the second, third, and fourth order SOFI processing.

The enhancement of 2PE-ISM over 2P confocal and 2PE-ISM-SOFI over 2PE-ISM in these experiments is consistent with the measurement taken from the single quantum dot in Fig. 3. The enhancement of 2PE-ISM-SOFI3 over 2PE-ISM is observed to be 1.55×, which is lower than the expected value of 1.73; however, it is important to note that the analysis of the spots on the top row of Fig. 4 indicates that none of the spots is consisting of a single QD emitter. The enhancement of 2PE-ISM-SOFI4 over 2PE-ISM in the middle row images of Fig. 4 averages to a value of 2.1×, which slightly higher than the theoretically expected factor of 2 improvement. Higher order SOFI processing of the data shown in Fig. 3 is shown in Fig. S3 in the Supplementary Material. For the four most intense spots seen in the 2PE-ISM-SOFI4 image in Fig. 4, the average FWHM in the horizontal and vertical cross-sections was 128 and 126 nm, respectively. After deconvolution the FWHM was further reduced to ∼75  nm (see Fig. 4 and Fig. S3 in the Supplementary Material). The raw datasets in Fig. 4 consist of 1000 frames of 128×128  pixel images with 64  μs pixel dwell time.

### 3.4 Imaging a Biological Sample

**Fig. 5:**
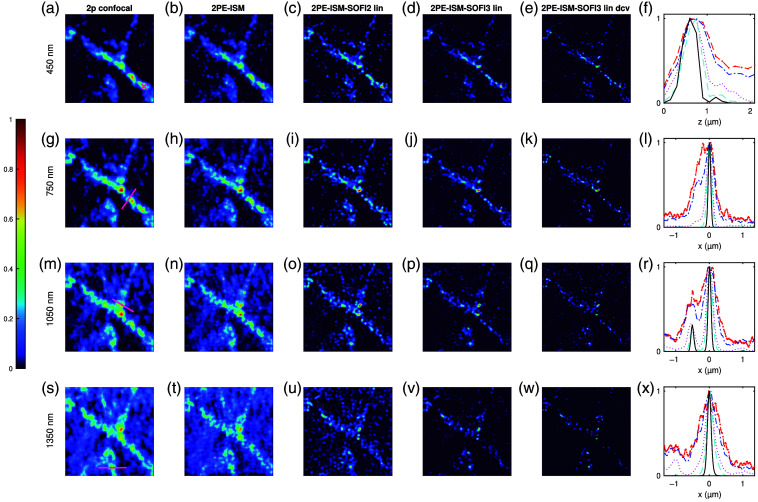
3D 2PE-ISM-SOFI imaging of tubulin labeled with QD625 in a mouse ventral midbrain neuron. Selected planes from the full stack are shown, with the distance from the first measured plane indicated. Dimensions: x and y – 10.9  μm. The brightest emitters are saturated in the image to allow better visualization of the less bright parts. The rightmost column shows an axial cross-section (f) and three lateral cross-sections (l,r,x), with the position of each indicated in the leftmost image (a,g,m,s). Color coding: red dots—confocal, dash-dotted blue—ISM, dotted magenta—ISM-SOFI2, dashed cyan—ISM-SOFI3, solid black—deconvolved ISM-SOFI3.

The applicability of 2PE-ISM-SOFI for 3D imaging of biological samples is demonstrated by imaging a sample of mouse brain cells, in which microtubules have been labeled with QD625 quantum dots. Mouse primary ventral midbrain neurons were cultured following the protocol used in Refs. 43–47. Wild-type C57BL/6 timed-pregnant adult female mice were obtained from the Texas A&M Institute for Genomic Medicine (TIGM), and animal handling was approved by the Texas A&M University Institutional Animal Care and Use Committee (IACUC, animal protocol number 2022-0252). Microglass coverslips (12 mm #1) (Electron Microscopy Sciences: Cat # 72231-01) for culturing neurons were coated and incubated individually for 1 h at 37°C with 100  μL of poly-L-lysine (Sigma-Aldrich: CAS. 25988-63-0) and poly-L-ornithine (Sigma-Aldrich: CAS. 27378-49-0), with Milli-Q^®^ water washes between each coating. Coverslips were then left overnight with 100  μL of laminin solution (10  μL of Laminin in 1 mL water) (Sigma-Aldrich: L2020-1MG) in 35×10  mm cell culture dishes (Corning^®^) according to IACUC regulations. Following preparation of glass coverslips for culturing neurons, the mouse ventral midbrain was dissected from ED14 embryos obtained from timed C57Bl6 timed pregnant mice euthanized with ${\mathrm{CO}}_{2}$ and cervical dislocation. The mouse embryonic midbrain was minced and further digested for 14 min in papain (Worthington Biomedical Corporation, Lakewood) solution at 37°C. Cell isolation was conducted by transferring brain bits in Deoxyribonuclease I (DNase I) (Sigma-Aldrich: CAS-No. 9003-98-9), followed by mechanical pipetting titration in 10% HS/PBS stop solution. Cells derived from mouse embryo midbrains were then seeded at a density of $3\times 10^{5}\text{ cells}/\text{coverslip}$ and maintained in prepared filtered neurobasal medium. Primary neuronal cultures obtained in this way were maintained in filtered Neurobasal + GlutaMAX medium containing GlutaMAX™-I (100×), B27-supplement, horse serum, and penicillin-streptomycin (ThermoFisher). Medium was further supplemented with the combination of ascorbic acid, kanamycin, and ampicillin obtained from Sigma-Aldrich. Primary midbrain neuronal cultures were immunostained following fixation under 10% formalin (VWR) for 30 min at room temperature. Cultures were washed with 1 × PBS (Thermo-Fisher), then permeabilized with 0.01% Triton X-100 (Sigma-Aldrich) in PBS for 2 min. Co-cultures were then blocked with 10% normal goat serum (NGS) (Abcam: AB7481-1002) in PBS and were followed by primary antibody incubation. Antibodies were prepared in 1% NGS with PBS solution for primary chicken anti-TH (Abcam: ab76442, 1:1000) and monoclonal rabbit anti-α-Tubulin antibody (Sigma-Aldrich: anti-TUBA4A (TUBA1) (T9026-.2 ML), 1:400) and incubated for 90 min. Secondary antibodies were prepared in 1% NGS with PBS solution for goat antichicken Alexa Fluor-488 (Abcam: ab150169, 1:1000) and donkey antirabbit polyclonal QD625 (Invitrogen: Q22806, 1:1000) and incubated for 60 min. Coverslips with DA cocultures were then washed with 1 × PBS and prepared for mounting. In Fig. 5, we show the results obtained for 2PE-ISM-SOFI (third order) of a 10.9×10.9×2.25  μm volume from this sample. Figure S4 in the Supplementary Material shows a comparison of 2PE-ISM with 2PE-ISM-SOFI (second order) for a different portion of the same sample. The data sets for Fig. 5 and Fig. S4 in the Supplementary Material, respectively, consist of 400 (for each of the 15 planes) and 1000 raw 256×256  pixel frames acquired with 64  μs pixel dwell time. Figure S5 in the Supplementary Material shows widefield SOFI imaging of a similar sample. The QD labeling of the cells is relatively sparse, which becomes increasingly evident with ISM, SOFI, and ISM-SOFI processing, and slightly noticeable in widefield, confocal, and 2P confocal images.

## 4 Discussion and Conclusion

In this work, we demonstrate the combination of 2PE-ISM with SOFI using a SPAD array, in which SOFI processing is applied after the ISM imaging reconstruction. In this work, we used self-blinking QDs for the experiments. Second-order SOFI processing allows to improve the resolution of 2PE-ISM by an additional factor of 1.4, and further improvement can be achieved using third- or fourth-order SOFI processing and deconvolution. With fourth-order SOFI postprocessing and deconvolution, we achieve a five-fold resolution (in the cross-section of the PSF) improvement over nondescanned 2PE microscopy from ∼370 to ∼75  nm. In the single-photon excitation demonstrations of SOFISM presented by Sroda et al.,^18^ a Fourier-reweighed fourth-order SOFISM enhancement of 4±0.7 was reported. Table 1 shows the resolution and relative improvement of the different processing steps.

**Table 1:** Resolution achieved with different individual and combined processing steps.

Imaging method	Resolution (nm)	Enhancement versus 2P confocal
2P confocal	375±15	—
+ deconvolution	264±5	1.42
2PE-ISM	272±5	1.38
+ deconvolution	196±2	1.91
2PE-SOFI (second order)	250±4	1.50
+ deconvolution	162±3	2.31
2PE-ISM-SOFI (second order)	192±7	1.95
+ deconvolution	127±5	2.95
2PE-ISM-SOFI (third order)	190±10	1.97
2PE-ISM-SOFI (fourth order)	126±8	2.98
+ deconvolution	75±5	5.00

The same group notes that even if a reasonable second-order was achieved for all their images, due to noise-sensitive estimation of the fourth-order cumulant, some data sets did not produce a reliable fourth-order SOFISM image, with the authors suggesting that this issue could arise from the switching time scales that can be on the order of the pixel dwell time.^18^ Indeed, it is known that QDs can exhibit switching from a bright on-state to a dark off-state with durations that can reach several seconds.

For the single-photon excitation using many diffraction-limited excitation foci referred to as iSIM SOFI, presented in Ref. 23, the authors explain that they could not reliably determine resolutions for third-order SOFI processing, due to fixed-pattern artifacts in the cumulant-processed images. For our 2PE-ISM-SOFI demonstration, we were able to successfully apply fourth-order SOFI analysis for images recorded using an excitation power of 6 mW before the microscope, and third-order SOFI analysis for images recorded with an excitation power of 12 mW before the microscope. Including our observation that 2PE-SOFISM suffers of artifacts due to long-lived dark states, this points at the possibility that relaxation from the dark state of QDs is suppressed by the infrared illumination, providing a means to control the on-off time ratio of the quantum dots. Our XC-SOFI approach that does not create a virtual pixel grid with smaller pixels may have alleviated some of the processing artifacts.^35^

The relatively sparse labeling of the biological samples is not optimal for quantitative analysis of the biological structure but enables the demonstration of the resolution improvement that 2PE-ISM-SOFI can achieve in the imaging of biological samples. It is important to note that the statistical approach of SOFI is quite robust with regards to label density, permitting multiple simultaneously fluorescing emitters in the PSF volume, and that SOFI is not restricted to QDs as in our demonstration. Other demonstrations such as SIM SOFI^23^ have been done using self-blinking dyes, such as Abberior FLIP 565 and custom-synthesized phalloidin-f-HM-SiR dyes. In future work, we also intend to investigate the applicability of alternative fluorophores, such as smaller polymer dots and self-blinking proteins such as Skylan-S,^48^ and study their fluorescence intermittency behavior under multiphoton excitation. For 2PE-SOFI, so far only QDs have been utilized,^30^^,^^49^ and the 2PE intermittency behavior of QDs has been studied in greater depth.^50^

Although we utilized a standard ISM reassignment approach, more sophisticated focus-ISM^51^ and super-resolution sectioning image scanning microscopy, dubbed $s^{2}\text{-}\mathrm{ISM}$^52^ approaches can increase reconstruction quality and boost the signal. In addition, these approaches boost axial resolution and out-of-focus light rejection. Assuming these better-quality ISM images, the second- and higher-order SOFI analysis would benefit as well.

The biological samples used in this work were a thin layer of fixed cells, but the applicability and advantage of multiphoton microscopy combined with ISM for in-depth super-resolution microscopy have been shown. Specifically, in a previous 3PE-ISM paper,^6^ detecting fluorescence with emission wavelength around 450 nm, we have demonstrated resolution enhancement at over 100  μm imaging depth into tissues, using the same high-resolution 100×/1.45 NA oil immersion objective. Previous literature on already demonstrated approaches^28^^,^^29^ has equally reported resolution-enhanced imaging at depths over 100  μm and at penetration depths up to 500  μm. The addition of SOFI does not create any extra requirements, because it simply enhances the given resolutions from 2PE-ISM or 3PE-ISM. Adaptive optics, either through variable telescopes to adjust z-focus and compensate for spherical aberrations, or via deformable mirrors, can help to restore the detection PSF quality, which can boost capabilities. In future studies, we will combine the advantages of SOFI-based imaging and two-photon excitation to enable super-resolution imaging of subcellular organelles *in situ* in 40 to 200  μm thick tissue samples.

In summary, in this work, we present a methodology that enables the successful application of SOFI to point scanning 2PE microscopy, reduces computational artifacts and avoids driving the independently blinking emitters into the dark state and prevents sample damage. This shows the potential to expand this method to higher-order multiphoton microscopy such as 3PE microscopy.^6^ As we have observed limited QD blinking in our previous work on 3PE-ISM,^6^ we believe 3PE-ISM-SOFI is feasible with some efforts to increase the 3PE efficiency, e.g., through fluorophore design and optimization of laser parameters. We demonstrate 3D 2PE-ISM-SOFI using self-blinking QDs and a SPAD array, as a promising alternative for resolution enhancement, especially in thicker and scattering biological samples. We have obtained a resolution of 200 nm using second-order 2PE-ISM-SOFI, which is a factor 1.8 smaller than what is achieved with 2PE microscopy alone. Also, with fourth-order 2PE-ISM-SOFI we obtained a resolution and 125 nm, which is 3 times smaller than what is achieved with 2P microscopy alone. Deconvolution allows to achieve 120 nm resolution with second order 2PE-ISM-SOFI, and with fourth order 2PE-ISM-SOFI ∼75  nm, resolution is achieved. Hence, even the second-order analysis reaches an ∼3-fold resolution enhancement over two-photon alone. This is often the limit in practice, to avoid the need for more raw data frames and to avoid cusp and other artifacts.^19^

Considering the emission wavelength of 625 nm and an effective NA of 1.2 of our microscope objective, the 75 nm resolution, we achieve with deconvoluted fourth order 2PE-ISM-SOFI surpasses best possible diffraction limited resolution 3.5-fold. Further investigations into how laser powers and dwell times with two-photon excitation can affect SNR and blinking dynamics in different fluorophores could help improve signal and quality for higher-order cumulants offering new imaging alternatives for applications in in-vivo imaging. Exploiting higher order cumulants in combination with the use of more sophisticated reassignment approaches^51^^,^^52^ can help to improve the system spatial resolution and further close the gap with most common realizations of well-established super-resolution techniques such as STED, PALM, and STORM, which routinely can achieve between 20 and 50 nm lateral resolution.^53^^–^^55^

## Supplementary Material

10.1117/1.JBO.31.8.086501.s01

## Data Availability

All data in support of the findings of this paper are available within the article or as Supplementary Material.
