# Supplementary material for: Breaking the 100-nm resolution barrier with multiphoton microscopy using image scanning microscopy and optical fluctuation imaging
Source: J Biomed Opt. 2026 Aug 2;31(8):086501. doi: 10.1117/1.JBO.31.8.086501 (PMC13430961; doi:10.1117/1.JBO.31.8.086501)
Supplement: Supplementary file 1 [file JBO_031_086501_SD001.pdf]

BREAKING THE 100-NM RESOLUTION BARRIER WITH MULTIPHOTON MICROSCOPY USING IMAGE SCANNING MICROSCOPY AND OPTICAL FLUCTUATION IMAGING: SUPPLEMENTAL DOCUMENT

Simulation results:

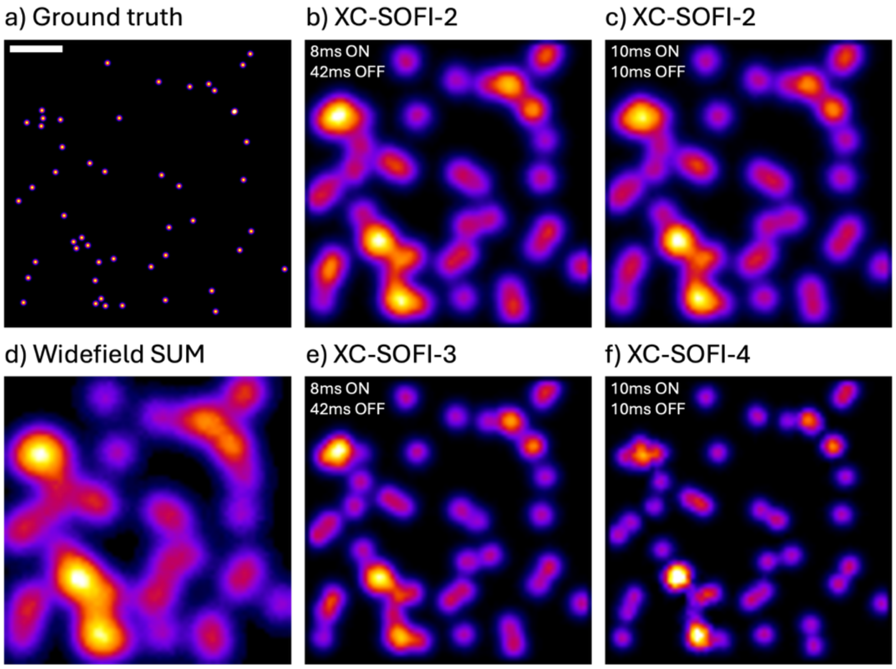

Fig. S1. Higher order SOFI with simulated dataset. (a) Arrangement of emitters used in the simulation. (b) 2nd order SOFI processed image when simulating data assuming 8 ms on- and 42 ms off-time. (c) 2nd order SOFI processed image when simulating data assuming 10 ms on- and off-time. (d) Sum of all simulated data frames, corresponding to the non-SOFI processed image. (e) 3rd order SOFI processed image when simulating data assuming 8 ms on- and 42 ms off-time. (f) 4th order SOFI processed image when simulating data assuming 10 ms on- and off-time. 3rd order SOFI processing simulated data assuming 10 ms on- and off-time results in a completely dark image. 4th order SOFI processing results in completely dark images for ON/OFF ratios of ~1:3.5 and ~3.5:1 [1].

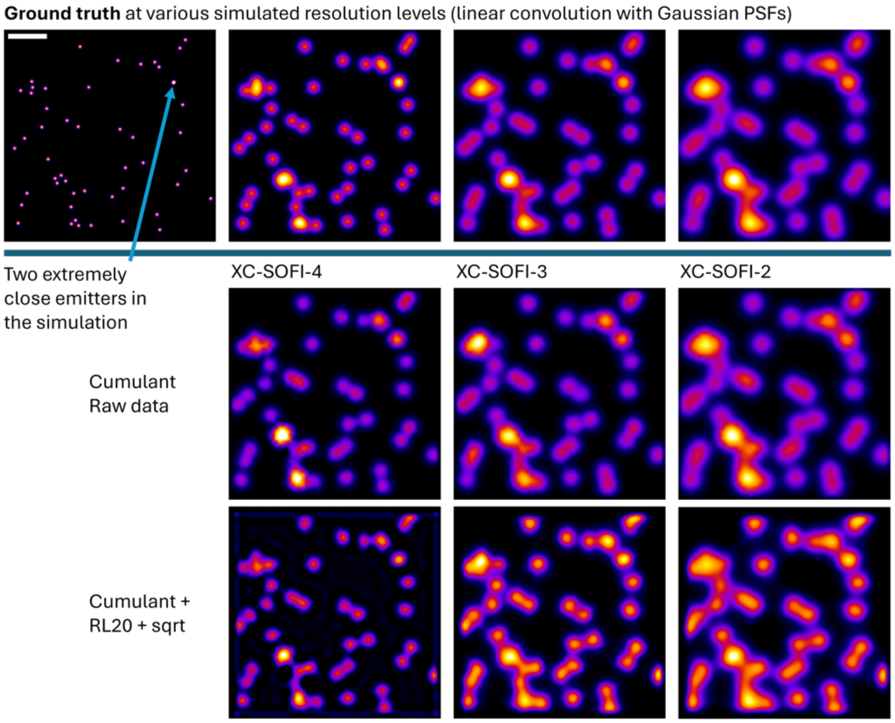

Fig. S2. Signal linearization of SOFI processed images on simulated data used for figure S1.

Additional experimental results.

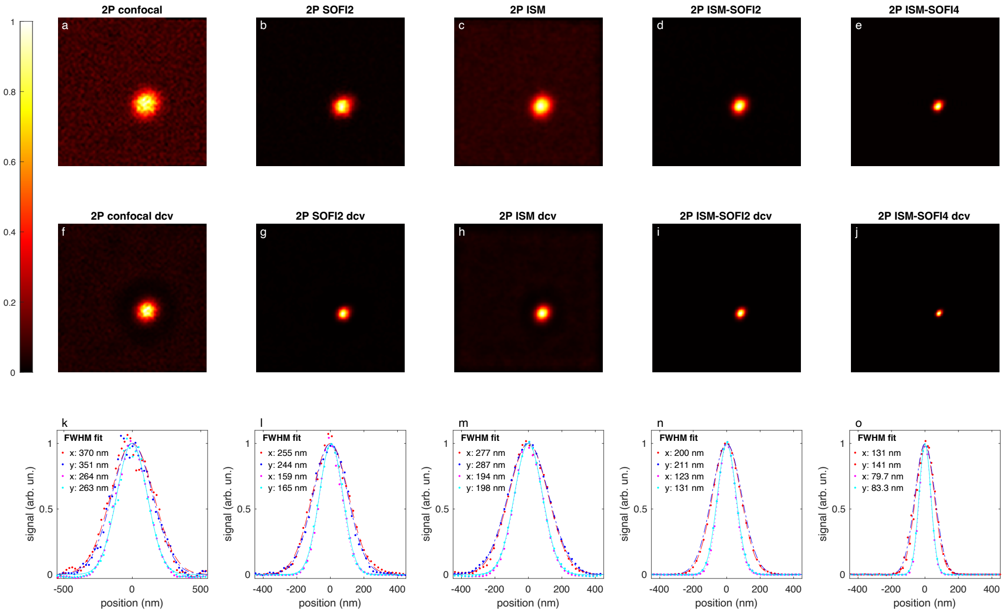

Fig. S3: Higher order SOFI processing and deconvolution of the data used for Fig. 3. Richardson-Lucy deconvolution (with the `deconvlucy()`-function in MATLAB) uses a calculated PSF that assumes the fitted FWHM widths from the ISM image, or it's square root for the confocal image). (a) 2P confocal. (b) 2P SOFI. (c) 2P ISM. (d) 2P ISM + SOFI. (e) 2P ISM + 4th order SOFI. (f)–(j) Richardson-Lucy deconvolved images corresponding to (a)–(e). For the ISM + SOFI processed image deconvolution is performed assuming the ISM PSF squared, and for the ISM + 4th order SOFI processed image deconvolution is performed assuming the ISM PSF to the 4th power. Panels (k)–(o) show the horizontal and vertical cross-section through the center of the bright spot in panels (a)–(e) in dashed red and dash-dotted blue respectively, and for the image in the panels (f)–(j) in dotted magenta and solid cyan respectively.

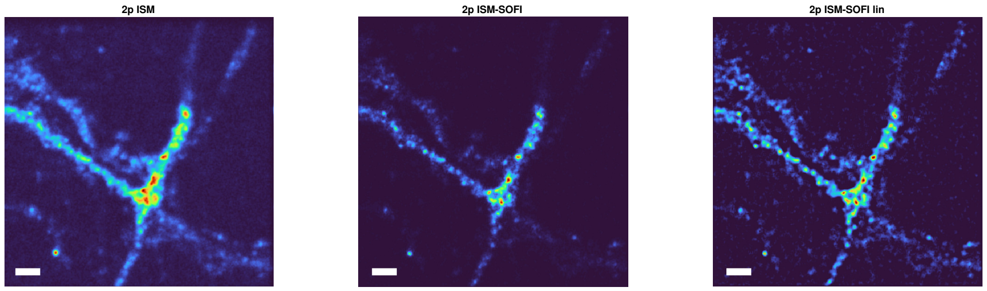

Fig. S4. Imaging of a mouse ventral midbrain neuron with QD625 labelled tubulin. Left panel: 2P ISM image after averaging 1000 image frames. Middle panel: 2P ISM 2nd order SOFI image obtained after SOFI post-processing the 1000 frames. Right panel: Linearized 2P ISM 2nd order SOFI image. The resolution enhancement and enhancement of the optical sectioning capabilities achieved with SOFI postprocessing are clearly visible. Scale bar 1 μm.

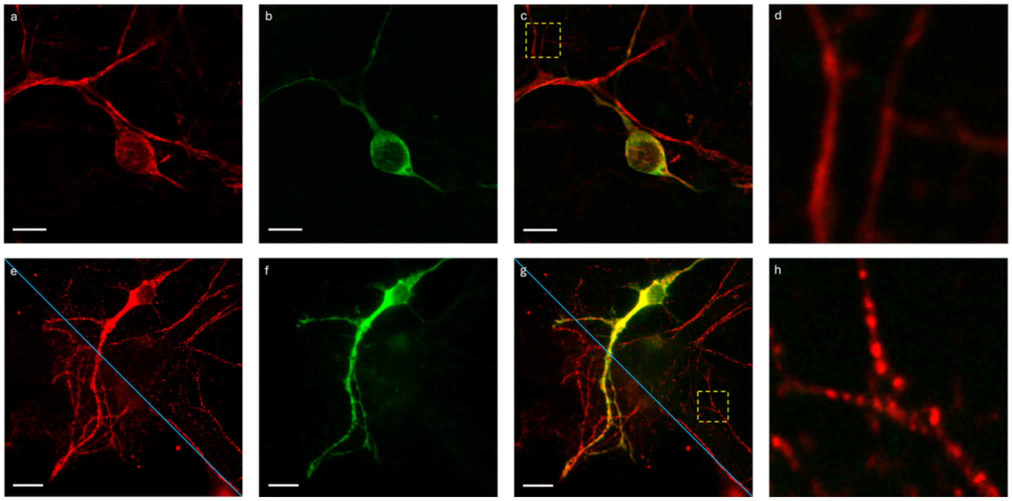

Fig. S5. Confocal and widefield imaging of mouse ventral midbrain neurons. (a-d) Confocal image with AlexaFluor647 (a, red) labelled tubulin and AlexaFluor488 (b, green) labelled tyrosine hydroxylase which is a marker for dopaminergic neurons. Panel (c) shows the overlay of both channels. Panel (d) shows a 10 µm x 10 µm zoomed region (location indicated by the yellow dashed square in panel (c)). Tubulin is observed with a resolution ~300 nm. (e-h) Widefield and SOFI image with QD625 (e, red) labelled tubulin and AlexaFluor488 (f, green) labelled tyrosine hydroxylase. Panel (g) shows the overlay of both channels. The lower left half of panels (e) and (f) show the average of 500 collected frames, the upper right half shows the SOFI processed result of the same 500 frames. Panel (h) shows a 10 µm x 10 µm zoomed region (location indicated by the yellow dashed region in panel (g). Tubulin is seen in red with a resolution of ~300 nm and ~200 nm, in the averaged and SOFI processed images respectively. Scale bars (a–c, d–g) 10 µm. The zoomed regions are chosen to show a similar structure as show in Fig. 5.

References

[1] A. Sroda, A. Makovski, R. Tenne, U. Rossman, G. Lubin, D. Oron, R. Lapkiewicz, "SOFISM: Super-resolution optical fluctuation image scanning microscopy: supplement," Optica 7, 1308 (2020), Supplement, https://doi.org/10.6084/m9.figshare.12863789
